# Patient‐Reported Outcomes and Esthetics of Single Dental Implants After 38.9 Years: A Cross‐Sectional Study

**DOI:** 10.1111/cid.70190

**Published:** 2026-10-21

**Authors:** Jan Kowar, Sargon Barkarmo

**Affiliations:** ^1^ Brånemark Clinic, Public Dental Health Care Service Gothenburg Sweden; ^2^ Department of Prosthodontics/Dental Material Science Institute of Odontology, Sahlgrenska Academy, University of Gothenburg Gothenburg Sweden

**Keywords:** cross‐sectional study, dental implants, esthetics, infraposition, long‐term follow‐up, OHIP‐14, patient‐reported outcomes

## Abstract

**Introduction:**

Long‐term evaluations of single dental implants have mainly focused on clinician‐reported outcomes, whereas patient‐reported and esthetic outcomes beyond 20 years remain sparsely documented.

**Objective:**

To assess patient‐reported outcomes, peri‐implant soft‐tissue esthetics, and crown infraposition of single dental implants after a mean follow‐up of 38.9 years.

**Materials and Methods:**

This cross‐sectional study included 13 patients with 18 single dental implants placed between 1982 and 1985 (mean follow‐up: 38.9 ± 0.8 years; range: 37.9–40.5 years). Patient‐reported outcomes were assessed using the Oral Health Impact Profile‐14 (OHIP‐14), the Orofacial Esthetic Scale (OES), and study‐specific Visual Analog Scale (VAS) evaluating overall treatment outcome, function, esthetics, and oral hygiene. Peri‐implant soft‐tissue esthetics were assessed using the Pink Esthetic Score (PES), with assessments conducted independently by two calibrated prosthodontists. Infraposition‐related incisal and cervical discrepancies were evaluated descriptively in six maxillary central‐incisor implant sites using intraoral scans or calibrated photographic analysis.

**Results:**

The mean OHIP‐14 score was 18.1 ± 7.3. Mean VAS scores were 92.8 ± 9.0 for overall treatment outcome, 95.5 ± 6.9 for function, 80.9 ± 27.0 for esthetics, and 90.2 ± 14.5 for oral hygiene. The mean total OES score was 40.8 ± 20.5 (*n* = 12; possible range: 0–80), with substantial interindividual variation. Mean total PES was 7.9 ± 2.2 and 8.6 ± 1.9 for the two observers. Interobserver reliability was assessed using the intraclass correlation coefficient (ICC), yielding an ICC of 0.47 (95% CI: 0.04–0.76). At all six assessable anterior implant sites, incisal and cervical discrepancies were observed, with the cervical discrepancy exceeding the incisal discrepancy in most cases.

**Conclusion:**

After a mean of 38.9 years in function, the available single‐implant cohort reported high overall and functional satisfaction, while patient‐reported esthetic outcomes and professional peri‐implant soft‐tissue assessments were more variable. Incisal and cervical discrepancies were present in the small assessable anterior subgroup; because baseline esthetic measurements were unavailable, these cross‐sectional discrepancies cannot be attributed specifically to progressive infraposition.

## 1 Introduction

In implant dentistry, single‐tooth replacements have traditionally been evaluated using clinician‐centered outcomes such as implant survival, bone level, peri‐implant tissue conditions, and technical complications. While these parameters remain essential, consensus recommendations in implant dentistry emphasize that patient‐reported outcome measures (PROMs) should complement clinical parameters when defining treatment success [1]. In parallel, an evidence‐based review has emphasized that the use and reporting of PROMs vary substantially across implant studies, which makes it difficult to compare results and draw overall conclusions across studies [2].

Esthetics is of particular relevance for single‐tooth implant reconstructions, especially in the anterior and premolar regions, where expectations may be high and minor discrepancies can be noticeable [3]. At the same time, patient‐perceived outcomes do not necessarily align with professional assessments, and high patient ratings may occur despite professionally identified esthetic shortcomings [4, 5]. Therefore, combining PROMs with professional esthetic assessments can provide a more complete picture of long‐term outcomes [3, 4].

Long‐term follow‐up is clinically important because changes can accumulate over decades, affecting both function and appearance. Aging‐related alterations in the dentition and surrounding tissues, restorative interventions, gradual soft‐tissue remodeling, and infraposition of implant‐supported crowns related to continued dentoalveolar changes can affect both function and appearance long after initial treatment [6, 7]. Even studies with longer observation periods rarely extend beyond two decades, although esthetic and soft‐tissue changes, including infraposition of implant‐supported crowns relative to adjacent teeth, may continue over time [6, 7, 8].

In a previous report, clinical and technical outcomes of single dental implants were evaluated over 38–40 years, showing favorable long‐term clinical outcomes [9]. However, long‐term data on patient‐reported outcomes and esthetic assessments from such extended observation periods remain limited. The present study builds on that long‐term material by adding validated patient‐reported outcomes and professional esthetic assessments and by assessing infraposition where applicable, thereby providing a complementary perspective on treatment outcomes at the same time point.

The aim of this cross‐sectional study was to assess patient‐reported outcomes, peri‐implant soft‐tissue esthetics, and crown infraposition after a mean follow‐up of 38.9 years.

## 2 Materials and Methods

### 2.1 Study Population

The present study was based on the same patient cohort as in a previous long‐term follow‐up study of single‐tooth implants treated at the Brånemark Clinic, Gothenburg, Sweden [9]. Briefly, the original cohort comprised 16 consecutively treated patients (8 females and 8 males), aged 14–48 years at the time of surgery, who received a total of 23 single‐tooth implants in the permanent dentition between 1982 and 1985. One patient with two implants had already been excluded at the 3‐year follow‐up after one implant failed 30 months after surgery and the second implant was recovered. Of the remaining 15 patients, 1 patient with 2 implants had died before the long‐term data retrieval, and 1 patient with 1 implant was excluded because the implant had become part of a larger implant‐supported bridge/prosthetic reconstruction. Thus, 13 patients with 18 implants were available for the present long‐term assessment. In the analyzed cohort, 54% were women; the mean age at implant surgery was 23.0 ± 5.35 years (range: 14–34 years), and the mean follow‐up was 38.9 ± 0.8 years (range: 37.9–40.5 years). Trauma accounted for 54% and aplasia for 46% of the indications. Sixteen of the 18 implants were located in the maxilla, and 72% replaced maxillary incisors (Table 1). The follow‐up examination included clinical examination, radiographic assessment, intraoral scanning, and clinical photography.

**TABLE 1:** Main characteristics of the 13 patients and 18 implants included in the long‐term analysis.

Patient	Sex	Age at surgery (years)	Follow‐up (years)	Indication	Implant position(s)
1	Male	33	38	Trauma	11
2	Male	21	40	Aplasia	22
3	Female	34	40	Trauma	21
4	Male	29	38	Trauma	21
5	Female	20	39	Trauma	11, 21
6	Female	17	39	Aplasia	34, 45
7	Female	20	39	Aplasia	23
8	Male	26	40	Aplasia	13, 23
9	Female	28	39	Trauma	21
10	Male	23	38	Aplasia	12, 22
11	Male	19	39	Aplasia	12, 22
12	Female	14	39	Trauma	11
13	Female	24	40	Trauma	21

*Note:* Patient numbering corresponds to the previously published clinical follow‐up study [9].

All data collection procedures were carried out in accordance with the 2013 revision of the 1964 Helsinki Declaration for biomedical research involving human subjects. The study was approved by the Swedish Ethics Review Authority (Dnr. 2023‐00888‐01). Written informed consent was obtained from all participants.

### 2.2 PROMs

PROMs were assessed using the Swedish versions of the Oral Health Impact Profile‐14 (OHIP‐14) and the Orofacial Esthetic Scale (OES), together with a study‐specific Visual Analog Scale (VAS).

The OHIP‐14 was used to assess oral health‐related quality of life during the preceding 3 months. The questionnaire comprises 14 items with 5 response categories coded from 1 to 5 (never = 1, seldom = 2, occasionally = 3, fairly often = 4, and very often = 5), yielding a possible total score of 14–70. The items cover difficulties in pronouncing words, impaired taste, oral pain, discomfort during eating, self‐consciousness, tension, dissatisfaction with diet, interrupted meals, difficulty relaxing, embarrassment, irritability, difficulty performing usual activities, reduced life satisfaction, and inability to function in daily life. Higher scores indicate poorer oral health‐related quality of life [10].

The OES was used to assess subjective perceptions of orofacial esthetics. The questionnaire comprises seven domain‐specific items and one overall item, each scored on a numerical scale from 0 to 10, where 0 represents the least favorable rating and 10 the most favorable rating. All eight items were summed to calculate the total OES score, yielding a possible range of 0–80. A “not applicable” option was available. The items address the appearance of the face, facial profile, mouth, dental arch, tooth shape, tooth color, gingival appearance, and the overall appearance of the face, mouth, and teeth. Higher scores indicate more favorable esthetic perceptions [11, 12].

In addition, four study‐specific VAS questions were used: “How do you rate the overall treatment outcome?,” “How satisfied are you with your treatment from a functional perspective?,” “How satisfied are you with the esthetic outcome of the implant crown(s)?,” and “How easy is it to maintain oral hygiene around the implant crown(s)?” Each response was recorded on a horizontal VAS anchored by a sad‐face symbol at the least favorable end and a happy‐face symbol at the most favorable end. Scores were transformed to a 0–100 scale, with higher scores indicating a more favorable patient‐reported outcome.

### 2.3 Esthetic and Infraposition Assessment

Peri‐implant soft‐tissue esthetics were assessed from clinical photographs using the Pink Esthetic Score (PES) according to Fürhauser et al. [13]. The PES comprises seven variables: mesial papilla, distal papilla, level of the soft‐tissue margin, soft‐tissue contour, alveolar process deficiency, soft‐tissue color, and soft‐tissue texture. Each variable is scored on a 0–1–2 scale, yielding a maximum total score of 14.

Before scoring, the two prosthodontists calibrated their assessments using the criteria and examples described by Fürhauser et al. [13]. The observers then scored all implant sites independently and were blinded to each other's scores. No repeated scoring session was performed; consequently, intra‐observer reliability could not be estimated.

Infraposition‐related discrepancies were assessed in patients treated with a single implant in a maxillary central‐incisor site. Six patients, corresponding to six implant sites, could be assessed. Intraoral digital scans were obtained using an intraoral scanner (CS 3700, Carestream Dental, Atlanta, GA, USA) and analyzed using the TRIOS Patient Monitoring tool (3Shape, Copenhagen, Denmark). Before measurement, the digital model was oriented using the occlusal plane as the horizontal reference. The incisal discrepancy was defined as the vertical difference between a horizontal reference line drawn from the incisal edge of the contralateral central incisor and the incisal edge of the implant‐supported crown. The cervical discrepancy was defined as the vertical difference between a horizontal reference line drawn from the cementoenamel junction of the contralateral central incisor and the marginal gingival level at the implant‐supported crown site. Both measurements were expressed in millimeters (Figure 1). The measurements were performed jointly by the two authors in a single measurement session. Because the model orientation was based on the occlusal plane rather than a prospectively standardized three‐dimensional coordinate system, some orientation‐related measurement uncertainty cannot be excluded.

**FIGURE 1:**
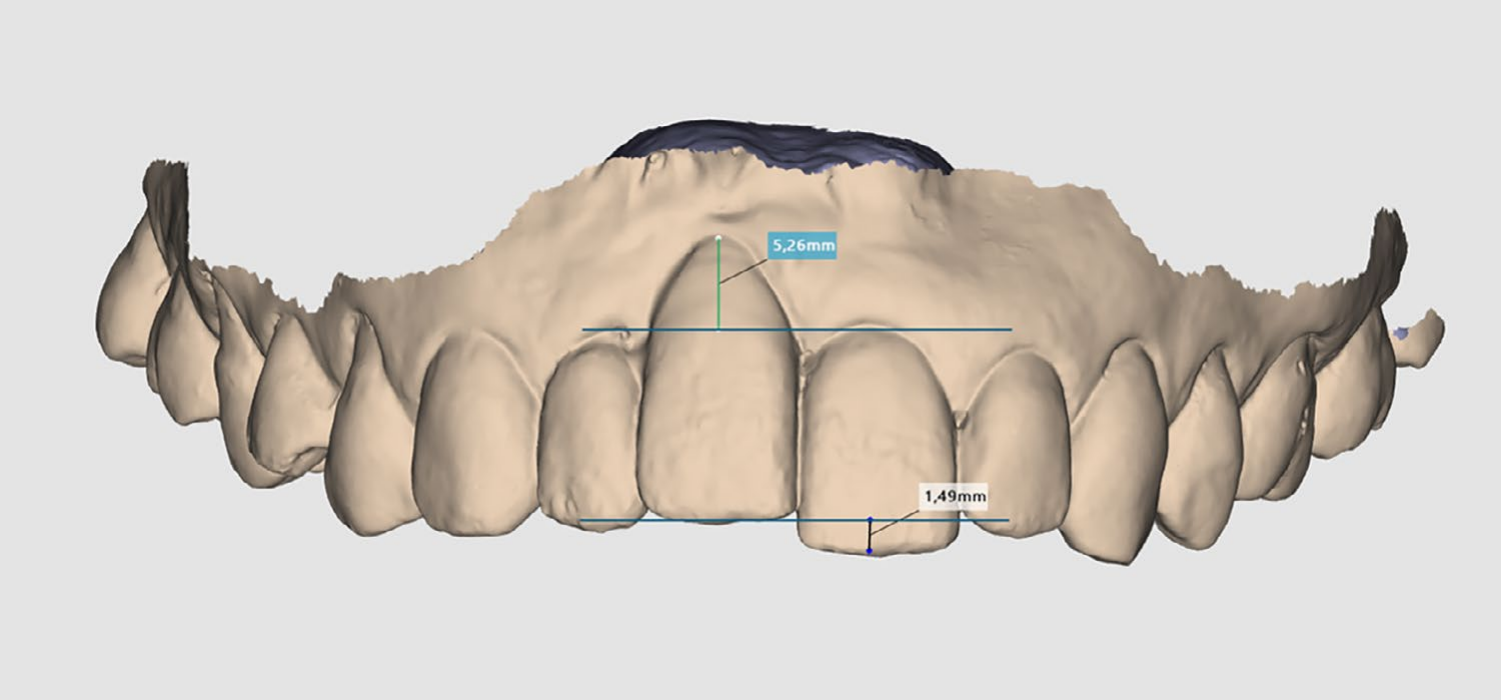
Measurement of incisal and cervical infraposition of the implant crown relative to the adjacent reference tooth.

For one patient, intraoral scan data were not available. In this case, the measurement was performed on a frontal clinical photograph using the image analysis software Fiji (ImageJ 1.54p, National Institutes of Health, Bethesda, MD, USA). The photograph had been taken straight from the front but had not been prospectively standardized to natural head position. The image was calibrated by determining the width of the central incisor from a periapical radiograph analyzed in Romexis (Planmeca, Helsinki, Finland), using the implant diameter as a reference for calibration. The calculated tooth width was then used to set the scale in Fiji, allowing the incisal and cervical discrepancies to be measured in millimeters using the same reference points. Potential perspective and projection error cannot therefore be excluded for this case.

### 2.4 Statistics

Descriptive statistics were used to summarize the data. Continuous variables were reported as mean and standard deviation (SD), together with median and range where appropriate.

PROMs, including the Oral Health Impact Profile (OHIP), the VAS domains assessing overall treatment outcome, functional satisfaction, esthetic satisfaction, and perceived ease of performing oral hygiene, as well as the OES, were analyzed descriptively.

Measurements of incisal and cervical infraposition relative to the adjacent reference tooth were also summarized using descriptive statistics.

Two prosthodontists independently evaluated the PES for all 18 implant sites. A paired *t*‐test was used only to assess whether a systematic mean difference was present between observers. Interobserver agreement for PES total scores was assessed using a two‐way random‐effects, absolute‐agreement, single‐measure intraclass correlation coefficient [ICC (2,1)] with a 95% confidence interval (CI). Infraposition‐related measurements were treated as descriptive/exploratory because of the small subgroup and the use of two measurement approaches.

Handling of correlated data: The 18 implants were clustered within 13 patients, with five patients contributing two implants. PROMs were analyzed at the patient level. Implant‐level PES data were summarized descriptively and used for the observer‐reliability analysis; no inferential comparisons between implant‐level clinical groups were performed. The limited sample size precluded multilevel modeling, and this clustering is considered when interpreting implant‐level descriptive findings. All statistical analyses were performed using IBM SPSS Statistics (version 29.0.2.0, IBM Corp., Armonk, NY, USA). Statistical significance was defined as *p* < 0.05.

The study was conducted and reported in accordance with the STROBE guidelines for observational studies.

## 3 Results

### 3.1 Patient‐Reported Outcomes

Patient‐reported outcomes were recorded using the OHIP‐14, four VAS domains, and the OES.

The mean OHIP‐14 score was 18.1 ± 7.3 (range: 14–39), indicating a generally low impact of oral conditions on quality of life. Patient satisfaction with the treatment outcome was overall high. The mean VAS score for the overall assessment of treatment outcome was 92.8 ± 9.0, while functional satisfaction was 95.5 ± 6.9. The mean score for esthetic satisfaction was 80.9 ± 27.0, showing considerably greater variability among patients. Perceived ease of performing oral hygiene around the implant crown was also rated highly, with a mean score of 90.2 ± 14.5.

The mean total OES score was 40.8 ± 20.5 (range: 8–66), based on responses from 12 of the 13 patients. One participant did not complete the OES. The wide range and SD indicate substantial interindividual variation in perceived orofacial esthetics (Table 2).

**TABLE 2:** Patient‐reported outcome measures.

Measure	*n*	Mean ± SD	Median	Min	Max
OHIP	13	18.1 ± 7.3	14.0	14	39
VAS‐1 overall outcome	13	92.8 ± 9.0	95.0	72	100
VAS‐2 functional satisfaction	13	95.5 ± 6.9	98.0	78	100
VAS‐3 esthetic satisfaction	13	80.9 ± 27.0	96.0	7	100
VAS‐4 oral hygiene	13	90.2 ± 14.5	96.0	48	100
OES total score	12	40.8 ± 20.5	47.5	8	66

*Note:* Descriptive statistics for Oral Health Impact Profile (OHIP), Visual Analog Scale (VAS) domains, and Orofacial Esthetic Scale (OES). Values are presented as mean ± SD with median and range.

### 3.2 PES

The peri‐implant soft‐tissue esthetics were assessed using the PES. Two prosthodontists independently evaluated all implant sites.

The mean total PES was 7.9 ± 2.2 for Observer 1 and 8.6 ± 1.9 for Observer 2. The scores ranged from 4 to 13 for Observer 1 and 5 to 12 for Observer 2.

The paired *t*‐test did not detect evidence of a systematic mean difference between the two observers (*p* = 0.19); this test was not interpreted as a measure of agreement. Interobserver agreement for total PES scores was ICC (2,1) = 0.47 (95% CI: 0.04–0.76), indicating limited‐to‐moderate agreement with considerable uncertainty.

### 3.3 Infraposition

Measurements of incisal and cervical discrepancies were possible in 6 of the 13 patients, corresponding to 6 maxillary central‐incisor implant sites. These measurements were analyzed descriptively because of the small subgroup and methodological limitations of the available reference systems.

Incisal and cervical discrepancies were measured relative to the contralateral central incisor. Cervical discrepancy was greater than incisal discrepancy in most cases (Table 3). Cervical discrepancy ranged from 0.69 to 5.26 mm, whereas incisal discrepancy ranged from 1.04 to 2.47 mm. Three implant crowns were original, and three had been remade. In four cases, the contralateral reference tooth was unrestored, whereas in two cases a porcelain restoration served as the reference. Age at implant placement ranged from 14 to 34 years among the six cases, as presented in Table 3. Notably, the largest cervical discrepancy (5.26 mm) occurred in the patient who was 14 years old at implant placement; given the descriptive sample of six cases, no association between age and discrepancy can be inferred.

**TABLE 3:** Incisal and cervical discrepancies relative to the contralateral reference tooth in the six assessable maxillary central‐incisor implant sites.

Tooth position	Reference tooth	Age at implant placement (years)	Incisal discrepancy (mm)	Cervical discrepancy (mm)	Implant crown	Reference tooth type
11	21	33	1.10	4.04	Original	Natural
21	11	34	2.47	3.19	Non‐original	Natural
21	11	29	1.34	2.70	Original	Natural
21	11	28	1.23	0.69	Original	Natural
11	21	14	1.49	5.26	Non‐original	Porcelain veneer
21	11	24	1.04	1.74	Non‐original	Porcelain crown

*Note:* Values are descriptive cross‐sectional measurements and should not be interpreted as longitudinal change from baseline.

## 4 Discussion

The present study provides patient‐reported and professional esthetic observations from an exceptionally long follow‐up of the earliest cohort of patients treated with Brånemark single‐tooth implants. After a mean of 38.9 years, overall and functional satisfaction remained high among the available participants, whereas esthetic outcomes were substantially more heterogeneous. The findings should therefore be interpreted as long‐term outcomes in a small surviving and available historical cohort rather than as estimates of contemporary treatment success. The combination of PROMs, peri‐implant soft‐tissue assessment, and descriptive incisal/cervical measurements provides complementary perspectives on how early single‐implant treatment may be perceived and appear almost four decades later.

Patient‐reported outcomes were generally favorable for function and overall treatment. OHIP‐14 scores were concentrated toward the lower end of the 14–70 scale, while VAS ratings for overall outcome, function, and oral hygiene were high. In contrast, esthetic VAS and OES results showed wide variation. The OES reflects overall orofacial appearance rather than the implant restoration alone, which should be considered when interpreting the mean score of 40.8 on a 0–80 scale. Previous implant studies have similarly reported that patient satisfaction and professional esthetic assessments do not necessarily coincide [4, 5, 14, 15, 16, 17, 18, 19]. This divergence may be particularly relevant after several decades, during which the natural dentition, restorations, facial tissues, and patient expectations may all change.

The professional assessment was intentionally focused on the peri‐implant soft tissues using the original seven‐variable PES described by Fürhauser et al. [13]. The later PES/WES approach described by Belser et al. [20] would have provided a broader standardized assessment of both pink and white esthetics; however, a WES assessment was not part of the original study protocol and was not added retrospectively. The PES results showed moderate mean scores but limited‐to‐moderate interobserver agreement (ICC: 0.47, 95% CI: 0.04–0.76), despite calibration and independent blinded scoring. This finding emphasizes the interpretive component of professional esthetic scoring and supports cautious interpretation of small differences in PES [21, 22]. Intra‐observer reliability could not be assessed because the photographs were scored only once, which is an additional limitation. Because the PES was originally developed for single‐implant assessment in the esthetic zone, its application to the few sites outside this region in the present historical cohort should be interpreted cautiously.

The historical treatment context is central to interpretation of the present esthetic findings. These implants were placed between 1982 and 1985, when single‐tooth implant treatment was still novel, and the surgical and prosthetic concepts differed substantially from contemporary esthetically driven implant therapy. The implants had machined surfaces, were treated with a two‐stage delayed‐loading protocol, and none of the patients received bone grafting before implant placement [9]. Early suprastructures included hexagonal interfaces, conical abutment cylinders, composite resin, and metal‐ceramic crowns, and several crowns were subsequently replaced for esthetic reasons [9]. Contemporary narrow‐platform and conical implant‐abutment designs, three‐dimensional prosthetically driven positioning, and routine hard‐ and soft‐tissue augmentation were not available in the same form. Consequently, the present peri‐implant tissue levels may reflect a combination of the original implant position, early component design and spatial constraints, subsequent tissue remodeling, prosthetic replacement, and long‐term dentoalveolar change rather than a single biological mechanism.

The six maxillary central‐incisor sites showed measurable incisal and cervical discrepancies relative to the contralateral tooth, with a larger cervical discrepancy in five of six cases (Figure 2). These findings are best regarded as descriptive cross‐sectional discrepancies consistent with soft‐tissue deficiency and/or crown infraposition, rather than definitive evidence of progressive infraposition over 38–40 years. No baseline esthetic measurements, standardized baseline photographs, or three‐dimensional records were available to establish when the discrepancies developed. Furthermore, three implant crowns had been remade, two reference teeth were restored, scan orientation was based on the occlusal plane, and one case was measured from a frontal photograph without prospectively standardized natural head position. Crown inclination and projection/orientation may therefore have influenced the measured values. Baseline implant depth is one plausible contributor to the cervical discrepancies, particularly because deep implant placement was observed in the previous clinical follow‐up [9], but this remains a hypothesis rather than a demonstrated causal explanation.

**FIGURE 2:**
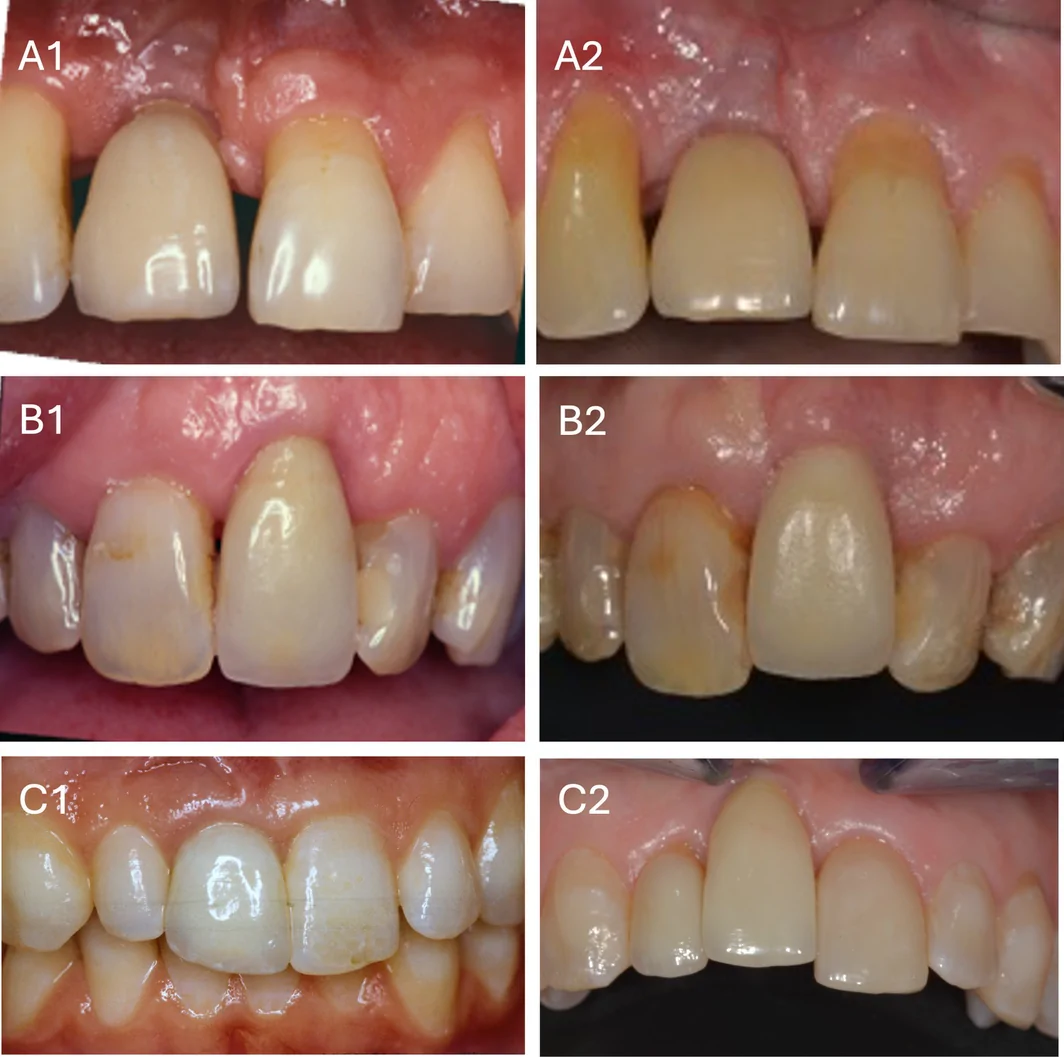
Long‐term changes in anterior dentition over approximately 40 years in three patients. (A1–C1) Baseline clinical photographs. (A2–C2) Corresponding follow‐up images after approximately 40 years.

Continued craniofacial and dentoalveolar development is another relevant consideration, particularly because patients in this historical cohort were treated at young ages. The six patients in the infraposition subgroup were 14–34 years old at implant placement. The largest cervical discrepancy (5.26 mm) was observed in the patient treated at 14 years of age. This isolated observation cannot establish an age effect, but it is consistent with previous evidence that ankylosed implant restorations may become increasingly displaced relative to adjacent teeth as dentoalveolar development continues [7, 23, 24]. Implant placement at 14 years in the anterior maxilla would generally be considered premature under contemporary treatment principles. Clinically, implant placement in growing patients should therefore be delayed until craniofacial and dentoalveolar growth is sufficiently complete whenever possible, and patients should be informed that positional discrepancies may still develop during adulthood. Long‐term planning should favor restorations that can be retrieved or replaced when necessary and should include continued monitoring of esthetic, biological, and technical outcomes.

The high patient satisfaction despite visible professional esthetic discrepancies also warrants cautious interpretation. Smile line or lip line was not systematically recorded, and it is therefore unknown whether the cervical discrepancies were visible during smiling. A low smile line could reduce the perceptual impact of gingival‐level discrepancies and may partly explain the divergence between patient and professional assessments, but this cannot be tested in the present dataset. Other important limitations include the small sample size, potential survivorship/selection bias after almost four decades, clustering of multiple implants within some patients, the cross‐sectional esthetic assessment, absence of baseline esthetic measurements, lack of WES and intra‐observer PES reliability, and the limited infraposition subgroup with mixed reference conditions and measurement approaches. Conversely, the exceptionally long follow‐up and linkage to previously reported clinical and technical outcomes from the same cohort are important strengths. The previous report showed that all 18 implants available at the long‐term examination remained in function, with stable marginal bone levels and few technical complications, although crown replacement was common [9]. Recent 25‐year data have likewise demonstrated that peri‐implant clinical conditions remain relevant over extended observation periods, underscoring the value of integrating biological, technical, esthetic, and patient‐reported domains when interpreting very long‐term implant outcomes [25].

## 5 Conclusions

After a mean follow‐up of 38.9 years, the available single‐implant cohort reported high overall and functional satisfaction, whereas patient‐reported esthetic outcomes and professional peri‐implant soft‐tissue assessments were more variable. In the small subgroup of maxillary central‐incisor implants, incisal and cervical discrepancies relative to the contralateral tooth were present at long‐term examination. Given the cross‐sectional design, absence of baseline esthetic measurements, and limitations of the available reference systems, these discrepancies cannot be attributed specifically to progressive infraposition. The findings highlight the value of combining patient‐reported and professional assessments when evaluating very long‐term implant outcomes.

## Author Contributions

**Jan Kowar:** concept/design, data collection, data analysis/interpretation, drafting the article, critical revision, approval. **Sargon Barkarmo:** concept/design, data collection, data analysis/interpretation, critical revision, approval.

## Funding

The authors have nothing to report.

## Conflicts of Interest

The authors declare no conflicts of interest.

## Data Availability

The data that support the findings of this study are available on request from the corresponding author. The data are not publicly available due to privacy or ethical restrictions.
